# The Morbid Conditions of the Blood

**Published:** 1863-12

**Authors:** Austin Flint


CHICAGO
MEDICAL JOURNAL.

VOL. VI.] DECEMBER, 1863. [No. 12.

### SELECTED.

### THE MORBID CONDITIONS OF THE BLOOD.

A Lecture by AUSTIN FLINT, M. D.

GENTLEMEN:—I have selected as the subject of the few lectures assigned to me during the preliminary term, the morbid conditions of the blood. I have selected this subject with reference, not so much to its attractiveness, as to its great importance. With our present pathological views, the question as to the existence of morbid conditions of the blood enters into the consideration of a very large proportion of diseases.

As expressive of the importance of the blood, it is distinguished as the *vital* fluid. In literature and common parlance it represents life. "Life's blood" is a common expression. To have one's blood is to take life. Its importance is shown by the fact that its presence in all the so-called vital organs is indispensable to the exercise of their functions. A striking and familiar illustration of this fact is afforded by the temporary loss of the mental faculties and consciousness, as a result of a momentary arrest of the supply of blood to the brain, in syncope or fainting. The blood, in fact, may be said to be the grand condition of vitality. Its detention from a part occasions the molecular death of the part, i.e. gangrene or sphacelus. Its abstraction, beyond a certain limit, from the body, occasions general or somatic death. The suspension of its distribution by an arrest of the heart's action for two or three minutes only, is fatal. It forms a vital medium for all the organs essential to life, on which they are dependent, as the body or the blood itself is dependent on the surrounding atmosphere. The physiological relations of the blood to the solid parts being so intimate, it might reasonably be expected *à priori*, that pathological changes in this fluid should give rise to corresponding morbid phenomena in the organs and tissues of the body. Observation shows this to be true. There are grounds for the belief that a large proportion of the morbid actions and changes which occur in the solid parts, are due to prior alterations in the blood. In many instances, as will be hereafter seen, the dependence of the former on the latter may be inferred, or rendered probable, although not demonstrable with our existing knowledge. Supplies for the growth and repair of all the body are contained in the blood. This fluid, therefore, represents, in its constituents, all the elements which enter into the composition of all the solid parts. It is taking but a step from the prosaic walks of scientific fact to the domain of fancy, to say that the blood is the solid body in a liquid state. "The blood is the centre round which the general metamorphosis of animal matter revolves, and in which it is perfected."* It might be rationally predicted that morbid alterations in its composition and distribution may lead to diseases seated in the solids, and this will be found to be the case. Another aspect foreshadowing the importance of the blood in its pathological relations, is its office as a reservoir for the accumulation of effete principles, the detritus of the tissues, which are to be eliminated by excretion. Here is a source of disease, as will be presently seen. Again, the physiological activity or mobility of the blood is very great. In this respect it is in striking contrast to the solid parts. It is the seat of unceasing changes, and yet, in health, maintains a uniform state as regards its organization and composition. New matter, derived from ingesta, is daily added in considerable quantity, and a proportionate amount is derived from the decomposition of the tissues. Portions are appropriated by the different structures. Other portions are secreted for various useful purposes in the economy. Other portions are thrown off or excreted. There is a constant interchange of the gaseous elements with the surrounding atmosphere by means of respiration and through the cutaneous surface. Thus, it is the

* Lehmann.

seat of constant and great changes, denoting wonderful activity, and yet its constitution remains the same. In this fact are admirably exemplified the precision and adaptation of the laws presiding over the safety and welfare of the organism. But this activity necessarily renders it more liable to morbid actions and conditions than the solid parts, which in health are less active and more stable.

The blood is a complex fluid. It contains a large number of ingredients, preserving, however, certain fixed anatomical character. Anatomically considered, it consists of certain corpuscular bodies, viz. the red globules, the white globules or leucocytes, and globulins, which are suspended in a liquid called the *liquor sanguinis*, blood-plasma, or intercellular fluid. These are resolvable, by analysis, into numerous elements, some of which are organic, i.e. peculiar to organic bodies, and others inorganic or mineral. Examples of the organic elements are, fibrin, albumen, hæmatine, etc. The inorganic elements embrace various saline ingredients, iron, water, and several gases. Further details belong to anatomy and physiology. It is necessary thus to glance at the composition of the blood, in order to arrange its morbid conditions. These con ditions relate to the different constituents of the blood; and, with a view to the consideration of pathological changes, the latter may be distributed into three groups. The first group will embrace the corpuscular, distinguished also as the organized, constituents. The second group will consist of the organic elements. The third group will comprise the mineral substances. Morbid conditions, affecting, severally, these three groups, will be first considered in the foregoing order, and afterwards, morbid conditions due to the introduction into the blood of substances which do not enter into its normal composition. Even with our present imperfect knowledge of the blood in health and disease, it is, in itself, a large field of study, which, considered as a distinct province of medicine, is called *hæmatology*.

Of the corpuscular or organized constituents of the blood, the most abundant and important are the *red globules*. The known morbid changes affecting these, relate in the first place to their number. They may be morbidly increased or diminished in number. An increase of the number of red globules beyond the healthy limit constitutes the morbid condition called *plethora*. A diminution below the limit of health constitutes the morbid condition called *anæmia*.

The relative proportion of the red globules to the other constituents of the blood may be increased by diminution of the latter. This obtains in a marked degree in epidemic cholera, owing to the draining away through the intestinal canal of the water of the blood, together with various elements held in solution by the transuded liquid. The density of the blood in this disease is notably increased; it becomes thick and heavy, and the circulation is mechanically impeded. Under these circumstances, the red globules are relatively in abnormal excess, although they are actually less in number than in health. The term plethora is only applicable to an actual increase of the number of red globules. This is now the significance of the term, without regard to the quantity of the mass of blood. An increase of the mass, causing over-repletion of the vessels does not constitute plethora, although implied in the etymology of the term. This condition is called *polyœmia.* Its existence to an extent sufficient to constitute a morbid condition of importance, is doubtful.

The functions of the red globules in health are not fully understood. Their relative normal ratio to the other constituents of the blood differs considerably in different animals, and they appear to sustain a certain relation to vigor, strength and activity; that is, they are abundant in races, breeds, and individuals, in proportion as the general attributes of the body just named are marked. Their importance is shown by the fact that animals bled nearly to death may be reanimated by injecting into the veins red corpuscles suspended in serum, and not by the introduction of the other constituents of the blood without the red corpuscles. From what is known of their physiological relations, it might be inferred that the effects of their morbid excess would be over-activity of the circulation and undue excitement of organs in proportion to their normal activity and the quantity of blood which they receive in health. The phenomena of plethora denote these effects. The power of the heart's action is increased. The temperature of the body is raised. The brain is stimulated, giving rise to unusual muscular mental energy and excitement. Sensibility and muscular irritability are augmented. In comparing, however, different persons, it is not easy to draw the line of demarcation between more or less intensity of the so-called sanguine temperament and plethora. A better idea of plethora, as a morbid condition, is formed by a comparison of the same person at different periods, and especially if the person have naturally a temperament not notably sanguine. He acquires more color in the prolabia and face. The mucous membranes are reddened. The pulse is full and strong. The heart's impulse is increased. The physical and mental powers are more active. The body is notably warm. Pain in the head is readily induced by stimulants or mental excitement, owing to the abnormal power of the circulation. This condition involves a liability to active cerebral congestion. It is supposed to constitute a predisposition to acute inflammations. It doubtless tends to render inflammations more intense, and to increase the symptomatic febrile movement. It may favor hæmorrhages, especially into the brain, by means of the increased force of the circulation. On the other hand, an abundance of red globules exempts from nervous disorders, to which, as will presently be seen, a paucity of red globules predisposes.

The causes of plethora are, first, a constitutional tendency, which may be congenital and inherited; second, overfeeding, with the use of generous wines and condiments; third, diminished expenditure of blood constituents in nutrition, incident to ease, idleness, and luxurious habits, the digestive and assimilative functions remaining active; and fourth, the arrest of periodical or habitual hæmorrhages, or some other drain to which the system had become accustomed. These several causes are frequently combined.

It is important for the physician to appreciate the condition of plethora, in order to avert the evils to which it tends, by appropriate management. And, as an incidental element in different diseases, it is to be taken into account in considering the effects of therapeutical measures. It is relieved for the time, most promptly and efficiently by bloodletting. An immediate effect of the abstraction of blood is a notable reduction in quantity of the red globules. Of course the propriety of resorting to bloodletting will depend on the degree of plethora and the apparent imminency of evil results. Other means to diminish the excess of red globules are, a reduced diet, as regards the quantity and quality of food, and exercise in order to increase the expenditure of blood-elements in repairing muscular waste, and render the amount of eliminated matter more abundant. Certain medicines appear to exert a direct effect upon the number of red globules. Mercury is such a remedy, as shown by the pallor which accompanies salivation. Mercurialization, however, is never indicated for the attainment merely of this object.

It is important not to confound plethora with other morbid conditions of the blood or circulation. Fulness of the vessels due to some impediment to the circulation, has not unfrequently been considered as plethora. This may exist where the red globules are diminished, rather than increased. A pseudo-plethora, for example, is not uncommon in pregnancy, the red globules being diminished in this state. Bleeding was formerly employed with reference to this pseudo-plethoric condition of course with an injurious effect on the constitution of the blood. With pseudo-plethora, or fulness of the vessels, there is often evidence of deficient oxygenation of the blood, together with dulness and oppression instead of heightened activity of the functions of the brain and other organs. True plethora is to be determined by the symptomatic phenomena which have been mentioned, taken in connexion with the evidence afforded by the pulse and other symptoms of an unobstructed, free circulation, with the activity of the digestive and assimilative functions, aud the existence of one or more of the conditions under which this morbid condition is known to be produced. A microscopical examination of the blood may suffice to determine the existence of the plethora, if the observer be sufficiently practiced to decide whether the red globules in several successive specimens are in excess or not. It may be determined by quantitative analysis, but the process is too tedious and delicate for ordinary clinical purposes.

As regards the essential pathological nature of plethora, all that can be said is, it consists in a hypergenesis of the most important of the organized or corpuscular constituents of the blood, the red globules. The pathologist might expect to explain this morbid condition more fully, if the physiologist was able to tell us where and by what process the red globules are normally produced.

A morbid diminution of the red globules of the blood constitutes *anœmia*. The etymology denotes diminution of the mass of blood, but, conventionally, the term is used to signify reduction of the quantity of red globules. *Spanœmia* is sometimes used in the same sense.

The purest exemplification of anæmia is afforded by cases in which it has been produced by copious hæmorrhages or repeated bloodlettings. It is not easy to effect, except for a transient period, a considerable reduction in the mass of blood. After a loss by hæmorrhage or bloodletting, the quantity of liquid which has escaped is quickly replaced, but the red globules are not so speedily renewed,and hence the latter continue for a greater or less period to be deficient. This condition is one of the forms of so-called impoverished or poor blood. The degree of impoverishment varies. The proportion of red globules has been observed to fall below the normal range (120 to 130 in 1000 parts) to 70, 60, and even 21 to 1000 parts.

Anæmia is of frequent occurrence. It is incident to a variety of diseases. It gives rise to a multiplicity of phenomena. It is a condition highly important for the physician to appreciate and recognize. The knowledge of this condition obtained within late years constitutes one of the most striking of the characteristics of modern medicine in view of its importance on medical practice. It occurs much more frequently than the opposite morbid condition, viz.: plethora.

In general terms, the pathological effects of anæmia are the reverse of those due to plethora. The power of the circulation is diminished, and there is a deficiency of the functional energy of different organs, the more marked in proportion to the quantity of blood which they receive in health. The phenomena denote these effects. The animal temperature is lessened. Anæmic patients have coolness of the surface, and especially cold extremities. They are not so able to resist cold as the plethoric. The action of the heart is feeble; the pulse is small, weak, compressible. The action of the heart is easily disturbed, becoming rapid from slight causes, and frequently irregular. The mental energy is diminished; persons are not adequate to the intellectual efforts of which they are capable in health. The strength of will and determination of purpose are impaired. The vital functions are languidly performed. The muscular strength is diminished. The surface is pallid from the deficiency of the hæmatine or coloring matter contained in the red globules. This pallor is apparent in the face, and especially the prolabia. The mucous membranes accessible to view have less redness than in health. The countenance at once denotes the existence of anæmia if the condition be marked.

It induces a multiplicity of morbid phenomena arising from disordered action of the nervous system. The relations of th[illegible] blood to the functional activity of the nervous syst[illegible] kingly shown in the morbid phenomena [illegible] ter, which spring directly from morbi[illegible] And the special relations between t[illegible] nervous system are shown by the phen[illegible] mia. These phenomena are numerou[illegible] more frequent and prominent are as fol[illegible] sion, anxiety respecting health, hypocho[illegible]

per, want of buoyancy and energy, a feeling of lassitude, and a painful sense of inertia or indolence. There is apt to be a feeling of incapacity for muscular exertion greater than the actual loss of muscular power. The physical and mental powers are especially depressed during the process of digestion. Palpitations frequently occur, so that organic disease of the heart may be suspected by those not conversant with physical means of diagnosis, and is greatly feared by the patient. Neuralgia in various situations is apt to occur, and in females hyperæsthesia of the abdominal walls simulating peritonitis. The varied symptoms which have been heretofore described as belonging to spinal irritation are likely to occur in connexion with anæmia. It sustains a causative relation to nearly all the functional affections of the nervous system embraced under the head of the *neuroses.* A large proportion of persons affected with any one or more of this class of maladies are anæmic; and conversely, a large proportion of anæmic persons become affected with neurotic disorders. It is highly important that this pathological element be taken into account in the management of the neuroses. When it occurs independently of the various affections with which it is connected incidentally, it is characterized especially by phenomena relating to the nervous system. These phenomena may be said to constitute the pathological expression of this morbid condition of the blood.

If it be asked, what is the explanation of the occurrence of these phenomena in consequence of a diminution of the red globules, the pathologist can only say that he may hope to answer the question when the physiologist is able to explain the normal relation between the presence of the red globules and the functions of the nervous system. Pathological facts show that an essential relation does exist between these two anatomical elements of the body. The nervous system depends on this blood-constituent for the manifestations of healthy life, and hence a deficiency occasions manifestations of disordered life, or morbid vital phenomena.

The causes of anæmia, when it exists independently of the various affections with which it is associated, are frequently obvious, but in some instances not assignable. It is a result of hæmorrhages, from wounds, flooding after labor, and in cases of menorrhagia, or injudicious bloodletting. It may proceed from deficient alimentation; the food being insufficient in quantity or not sufficiently rich in alimentary principles. It is caused by a loss of certain of the elements of the liquor sanguinis or blood plasma, which are necessary to the production of red globules. Thus, frequent causes are prolonged lactation and a rapid succession of pregnancies. The obvious causes may be arranged into the three classes just stated, viz.: 1st, Causes which involve an actual loss of red globules, as in hæmorrhages; 2d, Causes involving a defective supply of materials for assimilation; and, 3d, Causes which occasion expenditure of the constituents of the liquor sanguinis on which the production of the red globules is dependent.

The causes are not always apparent. Anæmia is apt to occur in females at or near the age of puberty, where there has been no loss of blood, no dificiency in alimentary supplies, or no unusual expenditure of blood-plasma. Under these circumstances it constitutes the affection to which the name *chlorosis* was applied before the anæmic condition was fully understood. If this name be retained it should be considered as denoting anæmia under the circumstances just stated. It appears to be in some way connected with the evolution of the reproductive functions. In some cases it may be accounted for by the derangement of the assimilative functions at this period. In these cases the appetite is poor, the digestion disturbed, and there is apt to be a craving for indigestible, innutritive substances, such as chalk, slate, coal, etc. Addison has described cases of anæmia occurring without any obvious causation, accompanied by general debility, which progressively increases, at length ending fatally without appreciable lesions of any of the vital organs. Cases of this kind are occasionally met with, especially in hospital practice. Addison distinguishes them as cases of "idiopathic fatal anæmia." In a certain proportion of these cases, the surface of the body, to a greater or less extent, assumes a dark discoloration or a bronzed appearance, and in several successive cases the supra-renal capsules were found to be more or less disorganized. Addison inferred from these facts a pathological connexion between disease of the supra-renal capsule and the bronzed hue of the skin. Clinical observation, however, shows that the two events are not uniformly associated.

In a large proportion of the cases in which anæmia exists, it is incidental to, or a pathological element of some other affection. And, as thus associated, it may or may not claim the special attention of the practitioner. Of the great number of diseases in connexion with which it is connected either constantly or frequently, the following list will inclose the more prominent.

1. Tuberculosis. Anæmia is generally early developed in tuberculous affections, and may precede the deposit of tubercle.

2. Carcinoma. The pale, waxy, or straw-colored complexion which characterizes some cases of carcinomatous disease, denotes anæmia.

3. The affections embraced under the name of Bright's disease. Associated with œdema of the face, the pallid complexion of anæmia becomes quite characteristic of these affections. The blood-changes which belong to these affections (to be hereafter considered) lead to diminution of the red globules.

4. A host of affections which involve expenditure of other constituents of the blood than the corpuscles, i.e. constituents of the *liquor sanguinis*, such as chronic dysentery and diarrhœa, chronic pleurisy, purulent formations in any part of the body, leuchrrhœa, etc.

5. Affections which involvé loss of corpuscles, or hæmorrhage, as menorrhagia, hæmorrhoids, hæmatamesis, etc.

6. Affections compromising the assimilative functions by occasioning indigestion, vomiting, loss of appetite, etc.

7. Certain affections of the liver, and especially cirrhosis. It has been supposed that the red globules are produced within the liver. If this be true, diseases of this viscus may lead to their diminution by interfering with their production. But in cirrhosis this effect is due in a measure to the obstruction to the introduction of fresh alimentary supplies brought by the portal vein.

8. The periodical fevers, if protracted. The special cause of these fevers may induce anæmia even where the fevers are not developed. Persons inhabiting regions called miasmatic, are apt to become anæmic, although they do not experience fever.

Certain mineral substances introduced into the system lessen the red globules in a notable degree. This is true of lead. Anæmia is a pretty constant element of saturnine diseases; and it is observed in persons exposed to lead emanations before becoming affected with the characteristic diseases. The same is true of mercury. Mercurialization quickly reduces the quantity of red globules in a marked degree.

